# Antibody response and the clinical presentation of patients with COVID-19 in Croatia: the importance of a two-step testing approach

**DOI:** 10.1007/s10096-020-04019-y

**Published:** 2020-09-04

**Authors:** Oktavija Đaković Rode, Ivan-Christian Kurolt, Ivan Puljiz, Rok Čivljak, Nataša Cetinić Balent, Renata Laškaj, Mirjana Kujundžić Tiljak, Radojka Mikulić, Alemka Markotić

**Affiliations:** 1 University Hospital for Infectious Diseases “Dr. Fran Mihaljević”, Mirogojska 8, 10000 Zagreb, Croatia; 2 School of Dental Medicine, University of Zagreb, Zagreb, Croatia; 3 School of Medicine, University of Zagreb, Zagreb, Croatia; 4 Andrija Štampar School of Public Health, Zagreb, Croatia; 5 Catholic University of Croatia, Zagreb, Croatia; 6 Faculty of Medicine, University of Rijeka, Rijeka, Croatia

**Keywords:** COVID-19 diagnostics, Clinical and laboratory findings, Anti-SARS-CoV-2 antibody response, Serological methods, Two-step testing approach, Croatia

## Abstract

According to anti-SARS-CoV-2 seroresponse in patients with COVID-19 from Croatia, we emphasised the issue of different serological tests and need for combining diagnostic methods for COVID-19 diagnosis. Anti-SARS-CoV-2 IgA and IgG ELISA and IgM/IgG immunochromatographic assay (ICA) were used for testing 60 sera from 21 patients (6 with severe, 10 moderate, and 5 with mild disease). The main clinical, demographic, and haemato-biochemical data were analysed. The most common symptoms were cough (95.2%), fever (90.5%), and fatigue and shortness of breath (42.9%). Pulmonary opacities showed 76.2% of patients. Within the first 7 days of illness, seropositivity for ELISA IgA and IgG was 42.9% and 7.1%, and for ICA IgM and IgG 25% and 10.7%, respectively. From day 8 after onset, ELISA IgA and IgG seropositivity was 90.6% and 68.8%, and for ICA IgM and IgG 84.4% and 75%, respectively. In general, sensitivity for ELISA IgA and IgG was 68.3% and 40%, and for ICA IgM and IgG 56.7% and 45.0%, respectively. The anti-SARS-CoV-2 antibody distributions by each method were statistically different (ICA IgM vs. IgG, *p* = 0.016; ELISA IgG vs. IgA, *p* < 0.001). Antibody response in COVID-19 varies and depends on the time the serum is taken, on the severity of disease, and on the type of test used. IgM and IgA antibodies as early-stage disease markers are comparable, although they cannot replace each other. Simultaneous IgM/IgG/IgA anti-SARS-CoV-2 antibody testing followed by the confirmation of positive findings with another test in a two-tier testing is recommended.

## Introduction

At the end of 2019, a new severe respiratory infection caused by SARS-CoV-2 spread rapidly and resulted in a high mortality rate in Wuhan, China [1, 2]. In Croatia, this infection was soon recognised as a potential global threat, prompting the government and health officials to prepare a strategy for the emerging situation [3, 4]. Intensive epidemiological investigations were conducted in an attempt to contain the spread of SARS-CoV-2 infections. Active case finding and contact tracing coupled with the isolation of patients and the active health surveillance of their asymptomatic contacts were undertaken. Meanwhile, the number of COVID-19 cases was increasing dramatically in neighbouring Italy. Epidemiological measures included the strict control of state borders and entry into the country, the closing of schools and shops, and the reorganisation of healthcare services in preparation for the care of a large number of severe cases [3].

The first case of COVID-19 in Croatia was confirmed on 25 February 2020 [3–5]. Over the next 2 weeks, sporadic new cases were registered. Subsequently, the number of new cases recorded per day gradually increased and then stabilised at approximately 60 (from 37 to 96) new cases recorded per day from 21 March to 25 April. Comprehensive preventive measures provided good epidemiological control with a subsequent reduction in the number of new cases [3].

The clinical presentation of SARS-CoV-2 infection varied from asymptomatic and mild to severe and critical [1, 6–8]. Mild cases are not easily distinguishable from other respiratory tract infections as the first signs are similar. Early diagnosis and recognition of the disease are crucial for appropriate treatment and limiting viral spreading. During the pandemic, each patient with fever, cough, fatigue, shortness of breath, headache, sore throat, runny nose, or even diarrhoea should be managed as having COVID-19, and diagnosis can only be established with targeted microbiological diagnostics [2, 6–11]. Clinical assessment of the symptoms and signs in accordance with the epidemiological data and medical history determines which specimens are drawn for diagnostic procedures. The main diagnostic clinical samples are nasopharyngeal and/or oropharyngeal swabs, and in severe cases sputum, endotracheal, or bronchoalveolar aspirate. In patients with no respiratory symptoms, stool and feco-anal swabs could be used [7, 9, 12–14]. Molecular diagnostics is a mainstay of SARS-CoV-2 diagnosis. COVID-19 should be confirmed by the positive reverse transcriptase quantitative polymerase chain reaction (RT-qPCR) nucleic acid test for SARS-CoV-2. The success of RNA detection depends on the specimen and the time of sampling from symptom onset, viral load, medical skills, and a PCR procedure with a negligible false-negative risk [15, 16].

Serological diagnostics, as a complementary diagnostic procedure, can be helpful especially for delayed presentations and the retrospective diagnosis of mild cases [15–18]. The clinical value of antibodies in COVID-19 requires evaluation. There is as yet no gold standard for serological diagnostics, although investigations using different assays and methods are in progress [18]. The time for specific IgM, IgA, and IgG antibody appearance is presumed to be in accordance with the data for MERS and SARS. Antibodies can be expected in about 2 weeks after disease onset [19–21].

The aim of the present study is to describe the antibody kinetics in hospitalised patients with RT-qPCR-confirmed COVID-19, and analyse antibody response by using different serological methods, according to the clinical and laboratory data.

## Patients and methods

The study at the onset of pandemic enrolled 21 randomly selected hospitalised adult patients (aged 26–81 years) with laboratory-confirmed COVID-19, from whom 60 consecutive sera were analysed. COVID-19 diagnosis was defined by the use of RT-qPCR, which had been established at the Dr. Fran Mihaljević University Hospital for Infectious Diseases (UHID) in Zagreb almost a month before the first case was detected in Croatia [3, 5, 22]. The RNA from combined nasopharyngeal and oropharyngeal swabs in Hanks medium was isolated with the Roche Total Nucleic Acid Isolation kit on a Roche MagnaPure LC 2.0 (Roche, Germany). According to the WHO-recommended Charité protocol, utilising the E and RdRP gene targets on an Applied Biosystems 7500 real-time thermocycler (Applied Biosystems, USA), 5 μl of RNA was used for the detection of SARS-CoV-2 [22].

Blood samples for serology were collected at the first visit, and later in accordance with routine biochemical tests. All the samples were stored at − 20 °C until testing. The anti-SARS-CoV-2 IgA and IgG antibodies were tested using enzyme-linked immunosorbent assays (ELISA; Euroimmun, Germany) according to the manufacturer’s instructions. Briefly, ELISA is based on the recombinant SARS-CoV-2 structural S1 domain protein. The results were obtained in ratios, which serve as a relative measure for the concentration of antibodies in the serum. The antibody levels were determined by calculating the extinction ratio of the patient samples (S) over the cutoff calibrator value (CO; S/CO). For samples taken 10 days after onset, the manufacturer declared IgA and IgG sensitivity of 44.8% and 22.4%, and for samples taken after the tenth day of illness, sensitivity of 100% and 87.5%, respectively.

The same samples were also tested for anti-SARS-CoV-2 IgM and IgG with qualitative lateral flow immunochromatographic assay (ICA) (SARS-CoV-2 IgM/IgG Antibody Assay Kit by the Colloidal Gold Method, Maccura Biotechnology Co., Ltd.). Testing was performed according to the manufacturer’s recommendations. A clearly visible coloured quality control band and detection line, either IgG or IgM, were deemed positive for anti-SARS-CoV-2 antibodies. The final results were always read by two independent investigators and were considered as initial screenings that needed to be interpreted according to the clinical data.

Clinical, biochemical, and haematological data were obtained from the patients’ electronic medical records. We analysed the demographic data, clinical symptoms and signs, severity of disease, and laboratory and radiologic results.

The Ethical Committee of the UHID approved this study. Written informed consent was waived by the patients included.

### Statistical analysis

Descriptive statistical analysis was used. Absolute and relative frequencies for qualitative variables, median values, and interquartile range, as well as the mean and 95% confidence interval of the means, were calculated. The McNemar chi-square test was used to compare the differences between the two serological methods. Regression linear trends in IgA and IgG values that changed according to the severity of the disease are graphically presented.

## Results

### Demographic and clinical characteristics

We analysed 21 randomly selected hospitalised patients with RT-qPCR-confirmed SARS-CoV-2 infection who had blood drawn for serology. The demographic and main clinical characteristics of the COVID-19 patients are shown in Table 1. Out of 21 patients, 10 had comorbidities: 8 (38.1%) were older than 60 years, 3 (14.3%) had hypertension, 2 (9.5%) had diabetes mellitus and hypertension, another 2 (9.5%) had cardiovascular diseases, 1 (4.8%) had cerebrovascular disease and hypertension, and 1 had malignant disease. The main clinical laboratory findings are presented in Table 2.

**Table 1:** General characteristics and clinical findings in 21 patients with confirmed COVID-19

Patient characteristics	No. = 21 (%)
Age median (range), years	56 (26–81)
Male/female	13 (61.9)/8 (38.1)
Comorbidity	10 (47.6)
Mild disease	5 (23.8)
Moderate disease	10 (47.6)
Severe disease	6 (28.6)
Respiratory symptoms	
Cough	20 (95.2)
Fatigue	9 (42.9)
Shortness of breath	9 (42.9)
Sputum production	5 (23.8)
Sore throat	3 (14.3)
Nasal congestion	2 (9.5)
Systemic symptoms	
Fever (temperature > 38.0 °C)	19 (90.5)
Chills	8 (38.1)
Myalgia and arthralgia	7 (33.3)
Headache	5 (23.8)
Diarrheal	4 (19.1)
Nausea or vomiting	3 (14.3)
New loss of smell	1 (4.8)
Imaging	
Chest radiography	21 (100)
Chest computed tomography	3 (14.3)
Chest radiography abnormalities	16 (76.2)
Antiviral treatment	
Yes	17 (81.0)
Lopinavir/ritonavir	2 (9.5)
Hydroxychloroquine	6 (28.6)
Hydroxychloroquine plus azithromycin	9 (42.9)
No	4 (19.1)

**Table 2:** The main clinical laboratory findings in 21 COVID-19 patients

Laboratory data (reference range)	Findings
White cell count: median (IQR) × 10^9^ (3.4–9.7 × 10^9^)	5.7 (4.6–6.7)
Lymphocyte count: median (IQR) × 10^9^ (1.19–3.35 × 10^9^)	1.1 (0.8–1.4)
Lymphocyte relative percent: median (IQR) % (20–46%)	20.5 (13.4–26.4)
Aspartate aminotransferase (AST) U/L: median (IQR) (8–30 U/L)	36 (20–55)
Alanine aminotransferase (ALT) U/L: median (IQR) (10–36 U/L)	31 (16–70)
Lactate dehydrogenase U/L: median (IQR) (< 241 U/L)	227 (176–292)
Creatine kinase (CK) U/L: median (IQR) (< 153 U/L)	101 (36–163)
C-reactive protein mg/L: median (IQR) (< 5.0 mg/L)	25.2 (6.9–64.0)

### Serological results

A total of 60 consecutive sera were analysed for anti-SARS-CoV-2 antibodies. Eleven patients had 2 consecutive sera, 6 had 3 sera, and 3 had 4 sera and for one patient, 8 samples were tested. All samples were tested for IgA and IgG with ELISA, as well as IgM and IgG with ICA. Positive anti-SARS-CoV-2 antibodies according to the days after disease onset are presented in Table 3.

**Table 3:** Anti-SARS-CoV-2 antibodies tested with ELISA and ICA according to the days after disease onset in consecutive serum samples from the 21 patients with RT-qPCR-confirmed COVID-19

Days after onset	Samples, *N*	Anti-SARS-CoV-2 positive antibodies			
		ELISA		ICA	
		IgA, *N* (%)	IgG, *N* (%)	IgM, *N* (%)	IgG, *N* (%)
0–3	11	4 (36.4)	0 (0.0)	2 (18.2)	0 (0.0)
4–7	17	8 (47.1)	2 (11.8)	5 (29.4)	3 (17.7)
8–11	18	15 (83.3)	9 (50.0)	13 (72.2)	11 (61.1)
≥ 12	14	14 (100.0)	13 (92.9)	14 (100.0)	13 (92.9)
Total	60	41 (68.3)	24 (40.0)	34 (56.7)	27 (45.0)

*ELISA*, enzyme-linked immunosorbent assay; *ICA*, immunochromatographic assay

In the samples collected within the first 7 days of illness, anti-SARS-CoV-2 IgA and IgG tested with ELISA were found in 42.9% and 7.1%, respectively, while anti-SARS-CoV-2 IgM and IgG tested with ICA were detected in 25% and 10.7%, respectively. From day 8 after onset, ELISA anti-SARS-CoV-2 IgA and IgG were positive in 90.6% and 68.8%, while ICA IgM and IgG were positive in 84.4% and 75%, respectively. In general, sensitivity for ELISA IgA and IgG was 68.3% and 40%, respectively, while sensitivity for ICA IgM and IgG was 56.7% and 45.0%, respectively.

Seroprevalence in patients showed that, within the first 7 days after onset, only 5 (23.8%) had detectable IgM, while IgA was found in 9 (42.9%) patients. Anti-SARS-CoV-2 IgG was detected from day 8 in another 8 patients with ELISA and in 12 patients with ICA. In 4 patients, each consecutive serum was taken 6 or 7 days after the onset, and antibodies were negative for IgM/IgG ICA, although one patient had ELISA IgA on the sixth day of illness.

**Fig. 1:**
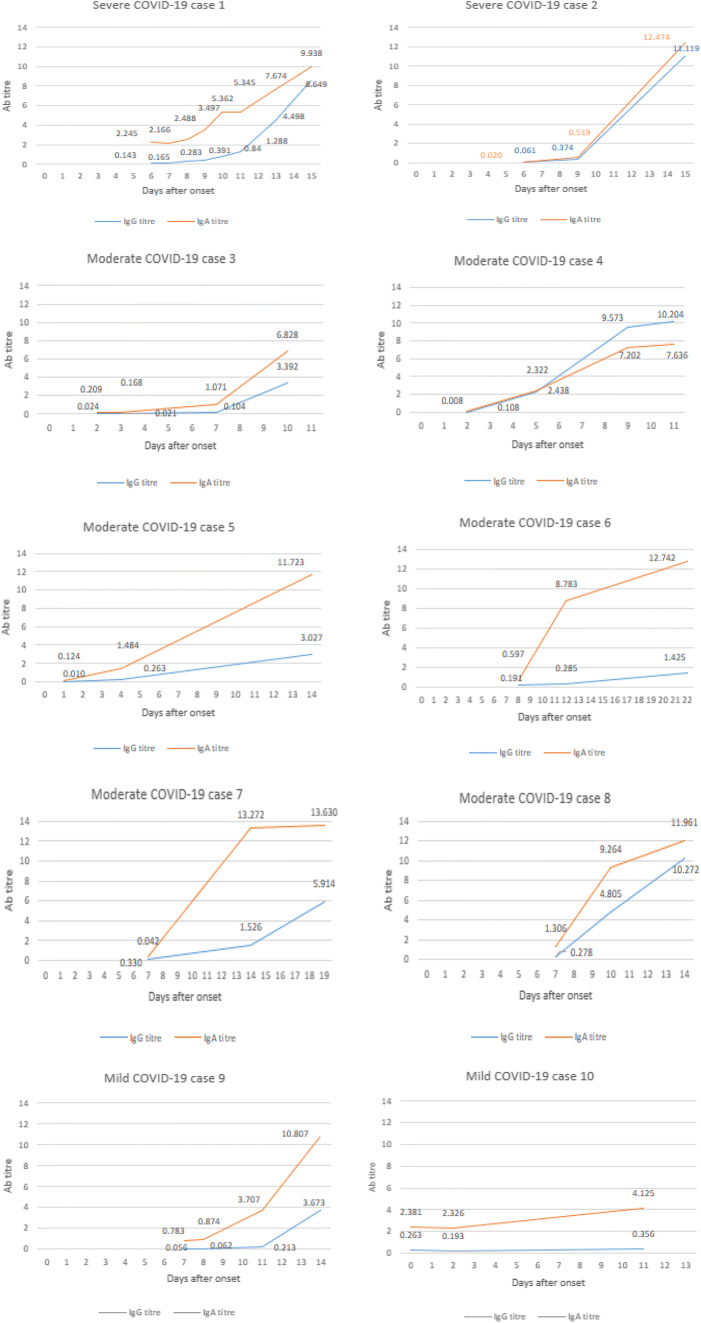
The kinetics of anti-SARS-CoV-2 IgG and IgA antibodies in 10 COVID-19 patients who had 3 or more follow-up sera drawn (Ab titre = antibody index serum/cutoff ratio; positive > 0.8)

The anti-SARS-CoV-2 IgG and IgA antibody kinetics of 10 patients who had 3 or more consecutive samples drawn are shown in Fig. 1. The results were presented as the antibody titre ratio (S/COV). A ratio higher than 0.8 is considered reactive for anti-SARS-CoV-2 antibodies. The mean ratio antibody titre for IgG was 2.3 (95% CI 2.9–4.2) and for IgA 4.7 (95% CI 4.0–5.8).

**Fig. 2:**
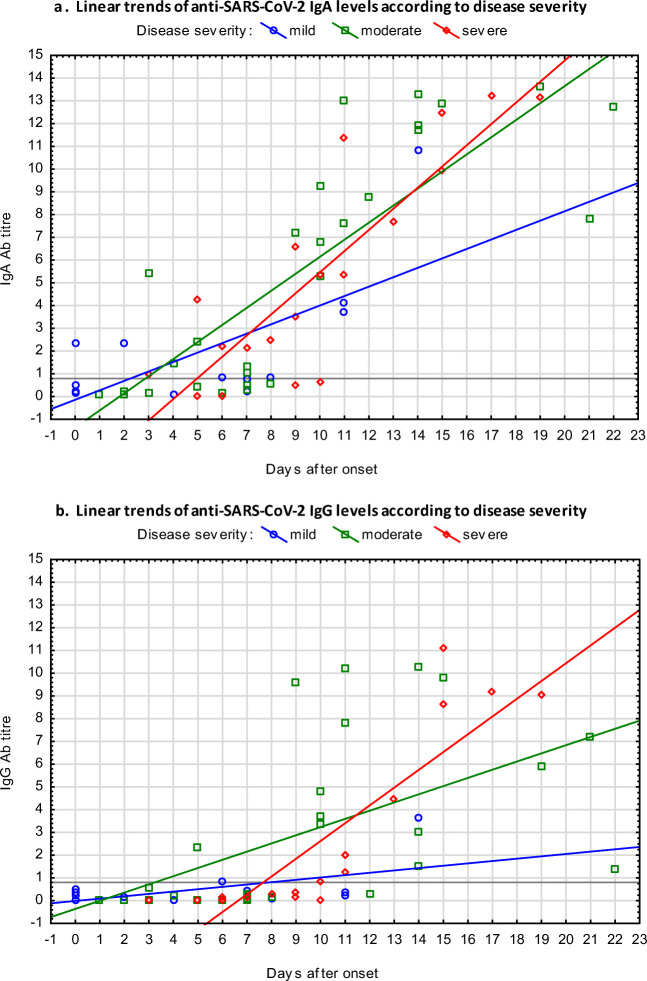
Linear trends of anti-SARS-CoV-2 IgA (scatterplot **a**) and IgG (scatterplot **b**) ELISA antibody levels according to disease severity (positive titre > 0.8)

Anti-SARS-CoV-2 IgA appeared earlier than IgG and reached higher antibody titres more rapidly. The longitudinal changes of antibody levels in patients are presented in Fig. 2. The linear trends of IgA were similar, independent of disease severity (Fig. 2a). Anti-SARS-CoV-2 IgG in severe cases reached a higher titre more rapidly than in mild cases (Fig. 2b).

No statistical differences were shown between ELISA and ICA (*p* = 0.092), between early antibodies anti-SARS-CoV-2 IgA ELISA and IgM ICA (*p* = 0.092), or between anti-SARS-CoV-2 IgG S1 antigen ELISA and IgG N/S antigen ICA (*p* = 0.453). Anti-SARS-CoV-2 IgG and IgA antibody titres were in high correlation (*p* < 0.05; *r* = 0.867). The positive and negative anti-SARS-CoV-2 antibody distributions determined by each method were statistically different (*p* = 0.016 between ICA IgM and IgG; *p* < 0.001 between ELISA IgG and IgA).

## Discussion

Since COVID-19 is a new disease, the antibody kinetics should be investigated [8, 11, 19–21, 23, 24]. As a contribution, we present the results of antibody dynamics in randomly selected hospitalised COVID-19 patients who were tested for anti-SARS-CoV-2 IgM and IgG with ICA and IgA and IgG with ELISA at the beginning of the pandemic. The study shows great variability in antibody response and emphasises the importance of follow-up serum testing, as well as the application of different serological methods as supportive diagnostic tools. Antibody detection depends on the time when the serum is drawn but also on the methods used, as well as individual host immunity. Average sensitivity of 68.3% and 40.0% for ELISA anti-SARS-CoV-2 IgA and IgG and 56.7% and 45% for ICA IgM and IgG, respectively, was found.

Anti-SARS-CoV-2 IgM and IgA were considered as equally important parameters for the early stage of the disease [11, 20]. During the first 7 days after onset, IgA was determined more frequently than IgM (42.9% vs. 25%). Furthermore, some patients had either IgM or IgA, suggesting the need to test both parameters at the same time. During the first 7 days of illness, simultaneously tested IgG was rarely detectable (7.1% and 10.7% by ELISA and ICA, respectively). Beyond day 8 after onset, anti-SARS-CoV-2 antibodies were detected in the majority of the patients: IgM and IgA in 84.4% and 90.6%, and IgG by ELISA and ICA in 68.8% and 75%, respectively.

The dynamics of IgA exhibited progressive linear trends and rapidly reached a higher titre than of IgG. The attacked respiratory mucous membranes generate a high amount of secretory anti-SARS-CoV-2 IgA antibodies early and induce strong mucosal immunity as a first-line barrier against the virus. A limitation was that IgA could not be compared with the quantitative IgM trend, since the ICA method is qualitative only. Anti-SARS-CoV-2 IgG appeared later than IgA and showed different linear progressive trends, depending on the disease severity. A positive correlation between the severity of the disease and IgG antibody levels was also reported by Zhao [19]. Accordingly, it could be a useful marker of COVID-19 progression. The role of IgG in long-term immunity needs to be further investigated.

Accurate COVID-19 diagnosis is a prerequisite for treatment and the implementation of epidemiological measures [8, 10, 25,26]. In Croatia, molecular testing with RT-qPCR was organised shortly after the first cases were confirmed in Europe [3–5, 22]. Combined nasopharyngeal and oropharyngeal swabs from all suspected COVID-19 patients were immediately tested, and positive RT-qPCR RNA confirmed the diagnosis. Although virus detection is considered fundamental, serological diagnostics have been introduced as a support for diagnosis. The risk of false-negative RT-PCR results is negligible, although possible faults must be considered even for sophisticated PCR due to various factors such as a low viral load in the upper respiratory tract, poor sampling techniques, sample quality, storage and transport conditions, and PCR reagent quality [7, 9, 11, 16, 26]. A combination of molecular and serological methods increases the likelihood of an accurate diagnosis, especially in a late phase of the disease [10–12, 26–29].

Serological diagnostics have limitations. For a correct diagnosis, the application of different tests and methods is recommended, due to the lack of a gold standard. Furthermore, knowing the specificity and sensitivity, as well as the positive and negative predictive values, is important in the interpretation of the significance of the results. Different antigens, as antibody catchers, may have an impact on the results, creating incomparable data for monitoring antibody dynamics or for seroprevalence studies. Our anti-SARS-CoV-2 IgA/IgG ELISA was based on the S1 antigen, and IgM/IgG ICA on the N and S protein fragments, but no statistically significant difference was found between these tests. However, the distributions of positive and negative findings for each method were significantly different, which suggests that, if only one method had been used, some of the subjects would not have been diagnosed correctly. All serological findings must be assessed in accordance with clinical and epidemiological data, taking into account the day of sampling and the gradual production of antibodies, as well as the different antibody classes detected with different methods [10, 11, 18–21].

Host immunity has an impact on antibody production [20–22]. From days 2 to 4 after the onset of the disease, 5 patients developed IgM/IgA while 2 others had no detectable antibodies until day 10, and seroconverted on day 12. Seroconversion can be expected during the second week of symptoms [11, 20–22, 24]. The reasons for delayed immune response may be immunosuppression, low dose of infectious virus, or alternative virus entry routes. The high antibody titre determined in more severe cases could be correlated with virus abundance. Increased antibody levels are not always accompanied by virus removal, suggesting that antibodies alone are not sufficient to clear the virus [2, 19, 21].

The significance of false-negative serological results in COVID-19 has been emphasised [10, 11, 20]. The incubation time in COVID-19 is too short for antibody development, so antibody detection is mainly unsuccessful when clinical symptoms are appearing. In some patients, antibodies can be detected as early as the fifth day of illness and the detection sensitivity increases after the eighth day of illness. For serological diagnosis of acute COVID-19, at least two serum samples should be tested. The first serum should be taken during the first physical examination, and consecutive ones at intervals of approximately 7 to 14 days.

Another problem is false positivity. For example, we reported on one patient with autoimmune hypergammaglobulinemia and respiratory symptoms, who was RT-qPCR-negative for SARS-CoV-2 in two separate samples and showed low anti-SARS-CoV-2 IgM reactivity but at the same time had detectable IgM against Lyme borreliosis, TBE, VZV, measles, and mumps. The similarity of coronaviruses may probably explain the false results of anti-SARS-CoV-2 IgG. Furthermore, false anti-SARS-CoV-2 IgA is also possible. In the evaluation of IgA assays, we encountered one acute EBV patient who had been diagnosed with IgA anti-SARS-CoV-2 reactivity prior to the COVID-19 pandemic. Although a limitation of this study is the small number of patients included, it is evidently important to detect the serological response in accordance with the duration and severity of the disease, the type of test, and the characteristics of the subjects. Testing consecutive samples and monitoring of the antibody kinetics are essential. Experience gained from low prevalence diseases points to the role of positive predictive values and the potential consequences of false-positive results.

In conclusion, antibody response in COVID-19 varies greatly and depends on the time the serum is taken and the severity of the disease but also on the type of test used. IgM and IgA antibodies as markers of early-stage disease are comparable, although they cannot replace each other. Simultaneous IgM/IgG/IgA antibody testing followed by the confirmation of anti-SARS-CoV-2 positive findings with another test in a two-tier testing approach is recommended. Even with the two-step testing approach, clinical interpretation is crucial for COVID-19 diagnosis.

## References

[1] Huang C, Wang Y, Li X, Ren L, Zhao J, Hu Y, et al. Clinical features of patients infected with 2019 novel coronavirus in Wuhan, China. Lancet 2020;395(10223):497–506 10.1016/S0140-6736(20)30183-5 PMC7159299 31986264

[2] Lu R, Zhao X, Li J, Niu P, Yang B, Wu H et al (2020) Genomic characterisation and epidemiology of 2019 novel coronavirus: implications for virus origins and receptor binding. Lancet [Internet] 395(10224):565–574 Available from: 10.1016/S0140-6736(20)30251-8 10.1016/S0140-6736(20)30251-8 PMC7159086 32007145

[3] No Title [Internet]. Available from: https://www.hzjz.hr/priopcenja-mediji/koronavirus-najnoviji-podatci/

[4] Čivljak R, Markotić A, Capak K. Earthquake in the time of COVID-19: the story from Croatia (CroVID-20). JOGH [Internet]. 2020;10(1). Available from: 010349 10.7189/jogh.10.010349 PMC7211414 32426118

[5] Čivljak R, Markotić A, Kuzman I (2020). The third coronavirus epidemic in the third millennium: what’s next?. Croat Med J.

[6] World Health Organization. WHO Clinical management of severe acute respiratory infection (SARI) when COVID-19 disease is suspected. Who [Internet]. 2020;2019(March):12. Available from: https://www.who.int/internal-publications-detail/clinical-management-of-severe-acute-respiratory-infection-when-novel-coronavirus-(ncov)-infection-is-suspected%0A. http://apps.who.int/iris/bitstream/10665/178529/1/WHO_MERS_Clinical_15.1_eng.pdf

[7] Wölfel R, Corman VM, Guggemos W, Seilmaier M, Zange S, Müller MA, et al. Virological assessment of hospitalized patients with COVID-2019. Nature. 2020 10.1038/s41586-020-2196-x 32235945

[8] European Centre for Disease Prevention and Control (2020) Pneumonia cases possibly associated with a novel coronavirus in Wuhan, China. ECDC: Stockholm

[9] Zhang W, Du RH, Li B, Zheng XS, Yang X, Lou HB (2020). Molecular and serological investigation of 2019-nCoV infected patients: implication of multiple shedding routes. Emerg Microbes Infect.

[10] Sethuraman N, Jeremiah SS, Ryo A. (2020) Interpreting diagnostic tests for SARS-CoV-2. Jama [Internet]. 2019:2019–21. Available from: http://www.ncbi.nlm.nih.gov/pubmed/32374370 10.1001/jama.2020.8259 32374370

[11] Di Giambenedetto S, Ciccullo A, Posteraro B, Lombardi F, Borghetti A, Sanguinetti M. Still much to learn about the diagnostic role of SARS-CoV-2 antibody detection [published online ahead of print, 2020 May 2]. Clin Infect Dis. 2020;ciaa532. doi:10.1093/cid/ciaa532. 10.1093/cid/ciaa532 PMC7197591 32358955

[12] Chen C, Gao G, Xu Y, Pu L, Wang Q, Wang L, et al. SARS-CoV-2–positive sputum and feces after conversion of pharyngeal samples in patients with COVID-19. Ann Intern Med. 2020;30ITC33-I2:M20–0991 10.7326/M20-0991 PMC7133055 32227141

[13] Yang Y, Yang M, Shen C, Wang F, Yuan J, Li J, et al. Evaluating the accuracy of different respiratory specimens in the laboratory diagnosis and monitoring the viral shedding of 2019-nCoV infections. medRxiv [Internet]. 2020;2020.02.11.20021493. Available from: http://medrxiv.org/content/early/2020/02/17/2020.02.11.20021493.abstract%0A. https://www.medrxiv.org/content/10.1101/2020.02.11.20021493v2

[14] Chen C-C, Chi C-Y. Biosafety in the preparation and processing of cytology specimens with potential coronavirus (COVID-19) infection: perspectives from Taiwan. Cancer Cytopathol [Internet]. 2020;1–8. Available from: http://www.ncbi.nlm.nih.gov/pubmed/32259402 10.1002/cncy.22280 PMC7262216 32259402

[15] To KKW, Tsang OTY, Leung WS, Tam AR, Wu TC, Lung DC et al (2020) Temporal profiles of viral load in posterior oropharyngeal saliva samples and serum antibody responses during infection by SARS-CoV-2: an observational cohort study. Lancet Infect Dis [Internet] 20(5):565–574 Available from: 10.1016/S1473-3099(20)30196-1 10.1016/S1473-3099(20)30196-1 PMC7158907 32213337

[16] Ai T, Yang Z, Xia L (2019). Correlation of chest CT and RT-PCR testing in coronavirus disease. Radiology..

[17] Abbasi J (2019). The promise and peril of antibody testing for COVID-19. JAMA - J Am Med Assoc.

[18] Amanat F, Nguyen T, Chromikova V, Strohmeier S, Stadlbauer D, Javier A, et al. (2020) A serological assay to detect SARS-CoV-2 seroconversion in humans. Nat Med. 2020.03.17.20037713 10.1038/s41591-020-0913-5 PMC8183627 32398876

[19] Zhao J, Yuan Q, Wang H, Liu W, Liao X, Su Y, et al. (2020) Antibody responses to SARS-CoV-2 in patients of novel coronavirus disease 2019. Clin Infect Dis 10.1093/cid/ciaa344 PMC7184337 32221519

[20] OKBA NMA, Muller MA, Li W, Wang C, GeurtsvanKessel CH, Corman VM, et al. (2020) SARS-CoV-2 specific antibody responses in COVID-19 patients. medRxiv [Internet]. 2020.03.18.20038059. Available from: https://www.medrxiv.org/content/10.1101/2020.03.18.20038059v1

[21] Guo L, Ren L, Yang S, Xiao M, Chang D, Yang F et al (2020) Profiling early humoral response to diagnose novel coronavirus disease (COVID-19). Clin Infect Dis:1–28 10.1093/cid/ciaa310 PMC7184472 32198501

[22] Corman VM, Landt O, Kaiser M, Molenkamp R, Meijer A, Chu DK (2020). Detection of 2019 -nCoV by RT-PCR. Euro Surveill.

[23] Petherick A (2020) Developing antibody tests for SARS-CoV-2. Lancet [Internet]. 395(10230):1101–1102 Available from: 10.1016/S0140-6736(20)30788-1 10.1016/S0140-6736(20)30788-1 PMC7270070 32247384

[24] Shi Y, Wang Y, Shao C, Huang J, Gan J, Huang X, et al. (2020) COVID-19 infection: the perspectives on immune responses. Cell Death Differ [Internet]. Available from: 10.1038/s41418-020-0530-3 10.1038/s41418-020-0530-3 PMC7091918 32205856

[25] van Doremalen N, Bushmaker T, Morris DH, Holbrook MG, Gamble A, Williamson BN (2020). Aerosol and surface stability of SARS-CoV-2 as compared with SARS-CoV-1. N Engl J Med.

[26] Ee S, Yong F, Anderson DE, Wei WE, Pang J, Chia WN et al (2020) Articles Connecting clusters of COVID-19: an epidemiological and serological investigation. Lancet Infect Dis [Internet]. 3099(20):1–7 Available from: 10.1016/S1473-3099(20)30273-5 10.1016/S1473-3099(20)30273-5 PMC7173813 32330439

[27] Bryan A, Pepper G, Wener MH, Fink SL, Morishima C, Chaudhary A, et al. (2020) Performance characteristics of the abbott architect SARS-CoV-2 IgG assay and seroprevalence in Boise, Idaho. J Clin Microbiol [Internet]. Available from: http://www.ncbi.nlm.nih.gov/pubmed/32381641 10.1128/JCM.00941-20 PMC7383515 32381641

[28] Li Z, Yi Y, Luo X, Xiong N, Liu Y, Li S et al (2020) Development and clinical application of a rapid IgM-IgG combined antibody test for SARS-CoV-2 infection diagnosis. J Med Virol:1–7 10.1002/jmv.25727 PMC7228300 32104917

[29] No Title [Internet]. Available from: https://www.who.int/emergencies/diseases/novel-coronavirus-2019/technical-guidance/naming-the-coronavirus-disease-(covid-2019)-and-the-virus-that-causes-it

